# Framingham Risk Score for Prediction of Cardiovascular Diseases: A Population-Based Study from Southern Europe

**DOI:** 10.1371/journal.pone.0073529

**Published:** 2013-09-05

**Authors:** Luis M. Artigao-Rodenas, Julio A. Carbayo-Herencia, Juan A. Divisón-Garrote, Vicente F. Gil-Guillén, Javier Massó-Orozco, Marta Simarro-Rueda, Francisca Molina-Escribano, Carlos Sanchis, Lucinio Carrión-Valero, Enrique López de Coca, David Caldevilla, Juan López-Abril, Concepción Carratalá-Munuera, Adriana Lopez-Pineda, on behalf of the Grupo de Enfermedades Vasculares de Albacete (GEVA)

**Affiliations:** 1 Community Medicine, Zone 3 Primary Health Care Centre, Albacete, Spain; 2 Medical Corps, Academia General del Aire, Murcia, Spain; 3 School of Medicine, University of Castilla-La Mancha, Albacete, Spain; 4 Community Medicine, Zone 2 Primary Health Care Centre, Albacete, Spain; 5 Clinical Medicine Department, Chair of Family, University of Miguel Hernandez, San Juan de Alicante, Spain; 6 Research Unit, Elda Hospital, Elda, Spain; 7 Community Medicine, Casas de Juan Núñez Primary Health Care Centre, Albacete, Spain; 8 Community Medicine, Zone 4 Primary Health Care Centre, Albacete, Spain; 9 Community Medicine, Casas Ibáñez Primary Health Care Centre, Albacete, Spain; 10 Community Medicine, Primary Health Care Centre, Valencia, Spain; 11 Internal Medicine, Primary Health Care Centre, Albacete, Spain; 12 Universitary Hospitalary Complex, Albacete, Spain; Brigham & Women's Hospital, and Harvard Medical School, United States of America

## Abstract

**Background:**

The question about what risk function should be used in primary prevention remains unanswered. The Framingham Study proposed a new algorithm based on three key ideas: use of the four risk factors with the most weight (cholesterol, blood pressure, diabetes and smoking), prediction of overall cardiovascular diseases and incorporating the concept of vascular age. The objective of this study was to apply this new function in a cohort of the general non Anglo-Saxon population, with a 10-year follow-up to determine its validity.

**Methods:**

The cohort was studied in 1992-94 and again in 2004-06. The sample comprised 959 randomly-selected persons, aged 30-74 years, who were representative of the population of Albacete, Spain. At the first examination cycle, needed data for the new function were collected and at the second examination, data on all events were recorded during the follow-up period. Discrimination was studied with ROC curves. Comparisons of prediction models and reality in tertiles (Hosmer-Lemeshow) were performed, and the individual survival functions were calculated.

**Results:**

The mean risks for women and men, respectively, were 11.3% and 19.7% and the areas under the ROC curve were 0.789 (95%CI, 0.716-0.863) and 0.780 (95%CI, 0.713-0.847) (P<0.001, both). Cardiovascular disease events occurred in the top risk tertiles. Of note were the negative predictive values in both sexes, and a good specificity in women (85.6%) and sensitivity in men (79.1%) when their risk for cardiovascular disease was high. This model overestimates the risk in older women and in middle-aged men. The cumulative probability of individual survival by tertiles was significant in both sexes (P<0.001).

**Conclusions:**

The results support the proposal for “reclassification” of Framingham. This study, with a few exceptions, passed the test of discrimination and calibration in a random sample of the general population from southern Europe.

## Introduction

Cardiovascular diseases (CVD) are widely accepted to be the most serious health care problem in both developed countries [1–3]. In addition, firm evidence supports the effectiveness of secondary prevention and there is almost unanimous agreement on its management [4–8]. However, the situation in primary care is not the same, particularly concerning the choice of methods for calculating or classifying cardiovascular risk (CVR) [9–16]. Guidelines for the prevention of CVD recommend the use of risk scores to identify adults at higher risk of CVD for whom preventive therapy has higher absolute benefits. Several scoring systems exist to help clinicians assess the 10-year CHD risk with the Framingham risk score the most widely used [17]. The Framingham-Study researchers [18] have therefore proposed a new model for use in the primary care setting, patently differentiated by gender. This “reclassification” of CVR is based on three basically sound ideas: first, use of just the four risk factors (RF) with the most weight as recognized in cardiovascular epidemiology: cholesterol, blood pressure (BP), diabetes mellitus and smoking; second, from the practical viewpoint, the physician is interested in determining the overall CVR, and the model therefore includes the prediction of all CVD events [coronary heart disease (CHD), cerebrovascular events (CVE), peripheral arterial disease (PAD) and heart failure (HF)], providing calibration factors for each entity that may be of interest to the physician; and third, inclusion of the concept of vascular age or heart age, and which can be calculated from the model.

There is reason to believe that the function, although practical, may not be applicable to the population of southern Europe or the Mediterranean, as is the population of Albacete (southeastern-Spain), where the prevalence of CHD (though not of other CVD) is lower than in northern, central and eastern Europe.

Marrugat et al. calibrated a Framingham-function, though in a population with a baseline coronary risk and one of the lowest incidences of CHD in the country. Their results suggest that this function underestimates the true risk in Spain [11]. The European alternative (SCORE) [9] is derived from a pool of various studies that are not strictly representative of the original population, and which excludes diabetic persons and morbidity, and which can only be used within the 40-65 year age group.

At the beginning of the 1990s (1992-1994), our research group studied a random stratified sample of the general population (N=1322) and calculated their risk for CHD using the classical Anderson-method [13] (as well as studying the prevalence of CVD and RF with their degrees of awareness, treatment and control) [19]. The aim of this present study was to “reclassify” this same cohort using the new proposal of D’Agostino [18] and determine their status after more than 10 years of follow-up. We would thus be able to assess, for the first time, to what extent this adapted function is valid in a strictly population-based study from southern-Europe with no Anglo-Saxon subjects.

## Methods

### Ethics Statement

The study was approved by the Healthcare Ethics Committee in the Albacete Health Region, in Spain. All study participants signed an informed consent document.

### Setting

This cohort study involved two examinations: the first one took place between 1992 and 1994 and the second between 2004 and 2006. The participants were examined at their own primary care centers and were selected from the general population aged >18 years in the province of Albacete, Spain.

### Selection and sample size

Our study sample comes from a cohort of the general population which was analyzed between 1992 and 1994 by our research team for a previous study [19–22]. This study comprised 1322 persons (69.5% response rate). The sample size of the current study is the same as the previous study, however, we selected the persons aged 30-74 years, as in the Framingham-study and other participants were excluded due to prior CVD or lack of laboratory results (N=872). The number of events of our study let us to estimate ROC curves of 0.80 or more with a standard error of 0.036 [23].

The sample was selected from the clinical records of primary care centers of Albacete by means of a two-stage sampling by conglomerates, stratified by size of population.

### Measurements

At the first examination cycle, the prevalence of CVD and cardiovascular RF was evaluated. Measurements of weight, height and BP were performed by GEVA group, as well as an electrocardiogram (ECG) and a Doppler ultrasound examination in order to calculate the ankle-brachial index were done. In addition, venous blood was drawn for analysis [19,21,22,24].

The second examination cycle, 10-14 years after, included study for the presence of CVD, including CHD [clinically recognized myocardial infarction (MI), ECG evidence of previous silent MI, clinically documented angina and death due to any CVD], as well as any type of CVE and PAD in the lower limbs with effect from grade II according to the Fontaine classification. Data were collected by the two trained doctors and data sources were: interview, medical records from hospitals and primary care centers, death certificates, direct contact with doctors and, in some isolated cases, direct contact with patient’s family. When the cause of death was uncertain, the cause specified by the family physician was accepted (two cases). Data were also recorded on personal history, a family history of early CVD and major RF and personal history of these same factors. Questions were also asked about toxic habits, nutrition and physical activity. Finally, weight, height, waist circumference, sagittal abdominal diameter and BP were measured, an ECG was done and venous blood was drawn.

### Measurement of the risk factors to calculate the CVR (first examination)

The BP was measured with a mercury sphygmomanometer in both arms, though we used the measurement of the left arm, like the Framingham-study, for comparative purposes. The subjects were seated and all the other conditions were as standardized. The use of antihypertensive medication was ascertained. The HDL-cholesterol was measured in the first examination by precipitation. All participants who smoked at least one cigarette per day were considered to be smokers. Diabetes was defined as fasting glucose >140 mg/dl and/or use of insulin or oral hypoglycemic medication.

### Follow-up events (second examination)

All manifestations of CVD between the initial examination (1992-94) and the second examination (2004-2006) were considered as outcome events. These events were as follows:

1. Any kind of clinically documented angina.
2. MI with a clinical report including enzyme activity, an ECG and/or isotope and/or angiographic study, or definitive determination with baseline ECG.
3. Stroke in the presence of permanent and objectified neurological deficit, or when neurological symptoms and signs were observed and resolved ad integrum and which the physician attributed to a transient ischemic attack.
4. PAD in the lower limbs if participants had Fontaine grade II to IV (grade I was excluded because its symptoms may not be related to CVD).
5. HF was only recorded if it was the cause of death.
6. Death due to CVD was considered if the specific cause of death on the death certificate was a CVD event, except those for whom the main cause of death was discordant or could not be fully explained by a CVD event. In doubtful cases, clinical records in hospitals or primary health centers were checked, and the physicians who attended the participants at the time of death were consulted, as well as the family if necessary.

### Statistical analysis

The data were analyzed using SPSS (SPSS for Windows, 15.0; SPSS Inc., Chicago,IL). The qualitative variables are expressed as the exact amount and percentage, the quantitative variables as the mean and standard deviation (SD). The association between qualitative variables was done with the chi-square test or Fisher’s test. Comparison between means was done with the Student t test for independent groups, or the Mann-Whitney U test if the conditions of normality using the Kolmogorov-Smirnoff or Shapiro Wilks tests were not met.

The individual risk was calculated with the D’Agostino-function, for men and women [18]. Its classification capacity in our population was confirmed with the measurement of the area under the ROC curve (discrimination). Calculations were then made of sensitivity, specificity, positive predictive value and negative predictive value for three different risk levels. The Hosmer–Lemeshow Χ^2^ statistic was used to evaluate the calibration or agreement between observed and predicted events and Mantel Haenszel test to evaluate the linear trend of the events in each of the three risk levels. All reported P values are two tailed.

## Results

**Figure 1:**
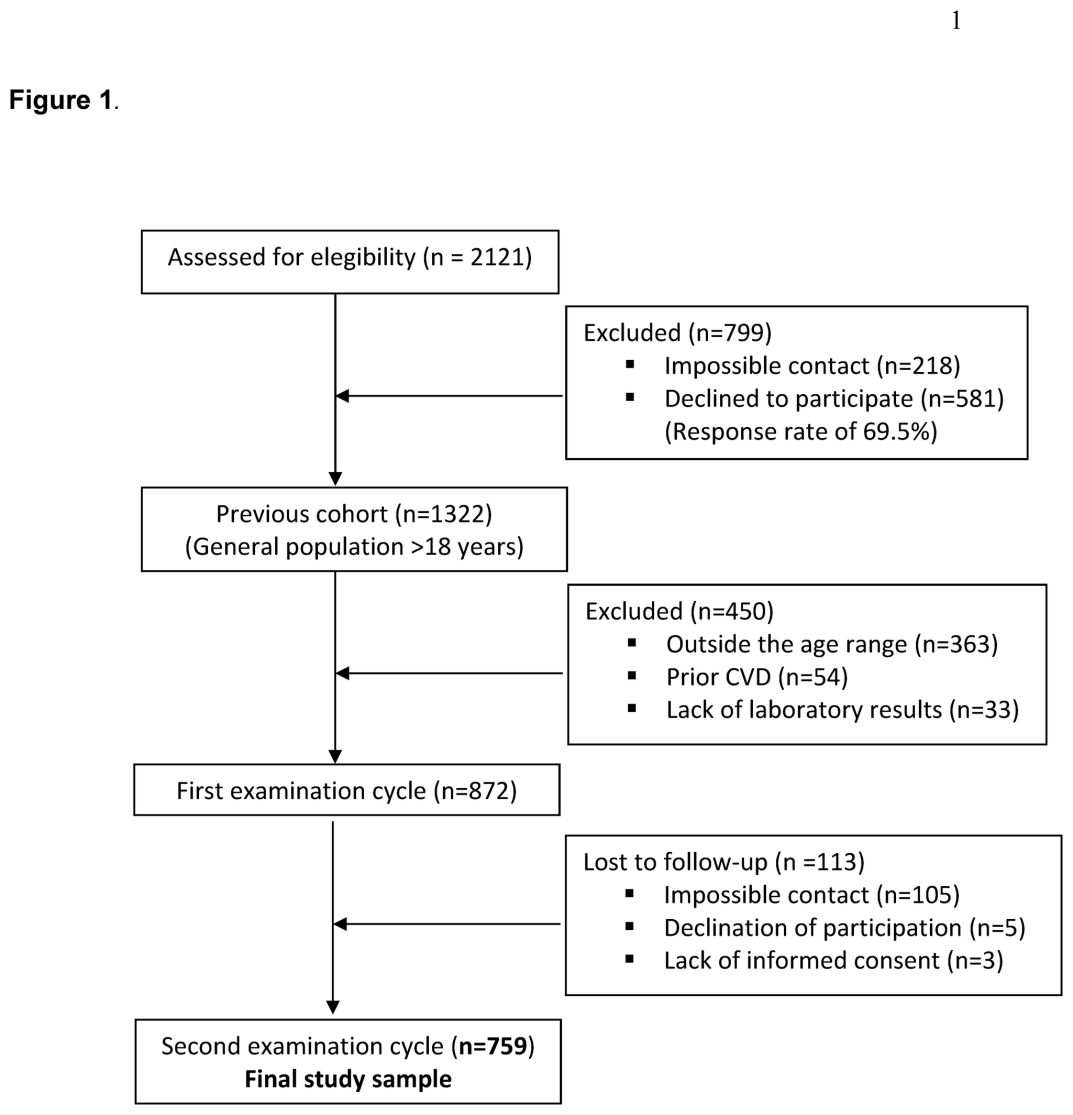
Flow of participants diagram.

As we have mentioned, our study sample comes from the cohort of previous study, where, initially, 2121 eligible patients were identified. Of these, 1903 were able to be contacted giving a response rate of 69.5%, thus, the cohort of our previous study [19–22] (1992-1994) comprised 1322 patients. However, in the current study 363 were excluded because they were outside the age range (30–74) and 54 were excluded for prior CVD (Framingham criteria). A further 33 were excluded due to lack of laboratory results, and, of the remaining 872 subjects, 113 (12.9%) failed to attend the second examination (2004-2006) due to one of three reasons: impossible contact (93%), declination of participation (4%) or lack of informed consent (3%). Thus, the final study sample included 759 persons (55.3% women) (See Figure 1).

Table 1 shows their baseline characteristics, also shows the corresponding baseline data for the participants in the Framingham-study which led to the new-model.

**Table 1:** Parameters used for the calculation of overall CVR and new events, according to sex, of the sample population versus the original population of the Framingham model.

**Characteristics**	**Women** Albacete **n = 420**	**Women** Framingham **n= 4522 (28% OFC)**	**P**	**Men** Albacete **n = 339**	**Men** Framingham **n= 3969 (22% OFC)**	**P**
**Age**, mean (SD)	51.6 (13.0)	49.1 (11.1)	**<0,001**	50.6 (12.9)	48.5(10.8)	**<0,01**
**TC,** mean (SD) mg/dL	203.4 (37.0)	215.1 (44.1)	**<0,001**	210.4 (37.2)	212.5 (39.3)	**NS**
**HDLc**, mean (SD) mg/dL	49.7 (12.1)	57.6 (15.3)	**<0,001**	43.0 (11.0)	44.9 (12.2)	**<0,01**
**SBP**, mean (SD) mm Hg	135.8 (23.3)	125.8 (20.0)	**<0,001**	133.0 (19.1)	129.7 (17.6)	**<0,01**
**Treated hypertension** n (%)	82 (19.5)	532 (11.8)	**<0,001**	35 (10.3)	402 (10.1)	**NS**
**Smokers**, n (%)	58 (13.8)	1548 (34.2)	**<0,001**	155 (45.7)	1398 (35.2)	**<0,001**
**Diabetes**, n (%)	47 (11.2)	170 (3.8)	**<0,001**	26 (7.7)	258 (6.5)	**NS**
**New CV events,** n (%)	31 (7.4)	456 (10.1)	**NS**	43 (12.7)	718 (18.1)	**<0,05**

**OFC=** Original Framingham cohort. **SD=** Standard deviation.

TC=Total cholesterol. **HDLc=** High-density-lipoprotein cholesterol.

**SBP=** Systolic blood pressure. **CV=** cardiovascular

**New CV events:** Coronary heart disease, cerebrovascular disease, peripheral arterial diseas, and heart failure. Albacete: coronary heart disease, cerebrovascular disease, peripheral arterial diseas, and lethal heart failure. Albacete excludes non-fatal heart failure

The mean follow-up of our cohort was 10.6 years (SD,2.3 years) and the mean overall risk for CVD, using the new function, was 19.7% (SD,15.7%) for men and 11.3% (SD,12.0%) for women.

### Discrimination of the “reclassification-model” in the sample population

**Figure 2:**
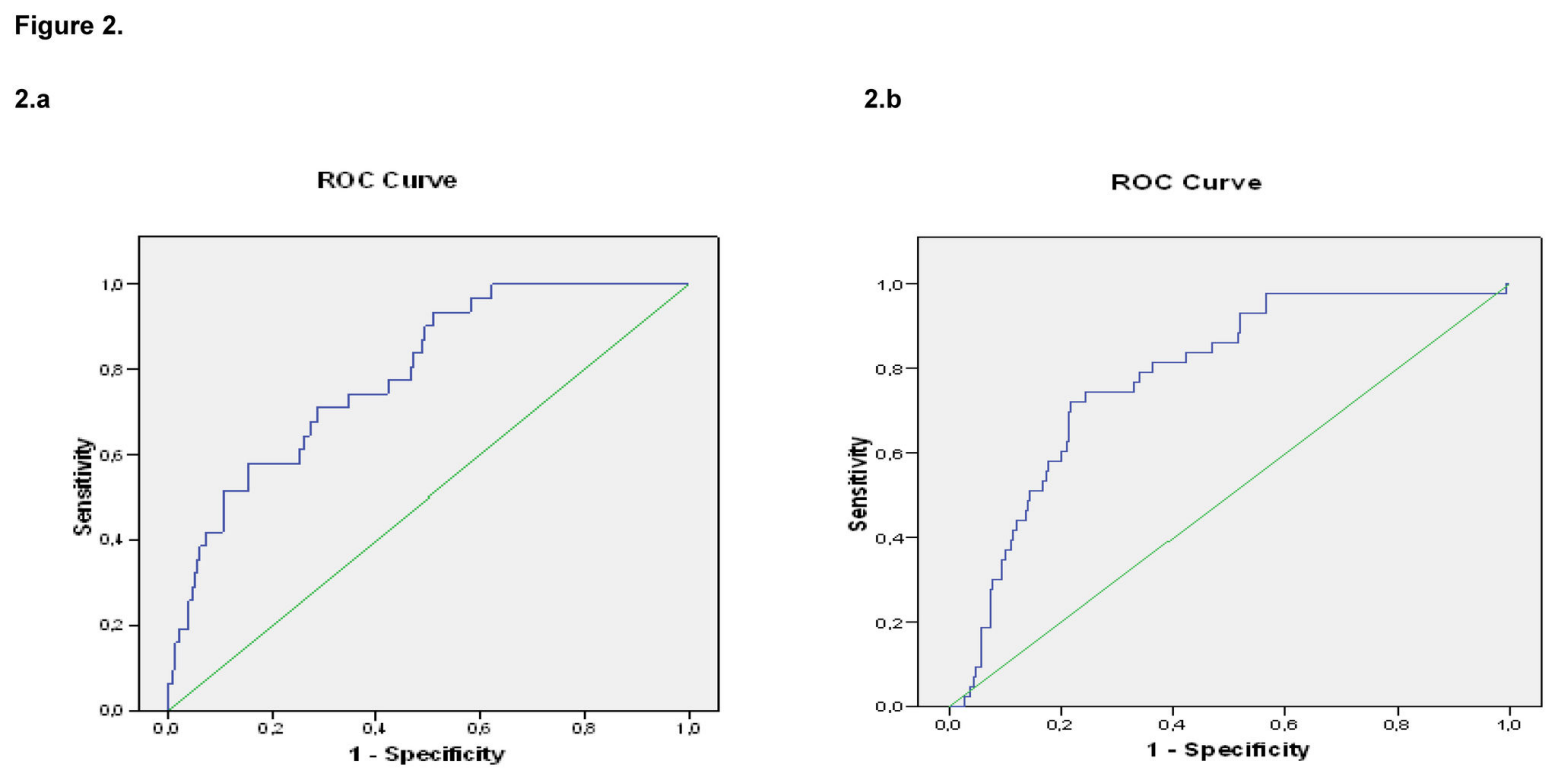
Area under the ROC curve for women (2.a) and men (2.b). The calculated cardiovascular risk correctly classified overall 78.9% (95%CI, 71.6-86.3) of the women and 78.0% (95%CI, 71.3-84.7) of the men (P<0.001 both).

The discriminative power (correct classification of those who had or did not have events according to the predicted overall CVR) was quantified by calculating the area under the ROC curve. The area under the global ROC was 79.4% (95%CI, 74.3-84.4). Figure 2 shows the results of this calculation for women (2a) and for men (2b). As is known, this ROC curve has different cut points, depending on whether we desire more sensitivity or more specificity in the test. Thus, the cut points for overall CVR that consider 75% specificity were: for men, 26.6% (74% sensitivity) and for women, 14.2% (61.3% sensitivity). On the other hand, the cut points that consider 75% sensitivity were: 21.1% overall CVR for men (67.7% specificity) and 8.6% for women (57.4% specificity). Table 2 shows the results in more detail.

**Table 2:** Sensitivity, specificity and predictive values for different levels of cardiovascular risk in women and men.

	**CVR <10%** (95% CI)		**CVR 10 and <20%** (95% CI)		**CVR > 20%** (95% CI)	
	Women	Men	Women	Men	Women	Men
**Se (%)**	25.8 (10.4/41.2)	2.3 (-2.2/6.8)	22.6 (7.9/37.3)	18.6 (6.9/30.2)	51.61 (34.0/69.2)	79.1 (66.9/91.2)
**Sp (%)**	38.1 (33.2/42.9)	58.4 (52.8/64.0)	76.4 (72.1/80.6)	75.7 (70.8/80.6)	85.6 (82.1/89.1)	65.9 (60.5/71.3)
**PPV (%)**	3.2 (1.0/5.4)	0.8 (-0.8/2.4)	7.1 (2.0/12.1)	10.0 (3.4/16.6)	22.2 (12.6/31.8)	25.2 (17.9/32.5)
**NPV (%)**	86.5 (81.4/91.6)	80.5 (75.2/85.8)	92.5 (89.6/95.4)	86.5 (82.3/90.6)	95.7 (93.6/97.8)	95.6 (92.8/98.4)

**CVR=** cardiovascular risk. **Se=** Sensitivity. **Sp=** Specificity.

**PPV=** Positive predictive value. **NPV=** Negative predictive value.

### Calibration. Agreement between predicted CVR and new events

**Figure 3:**
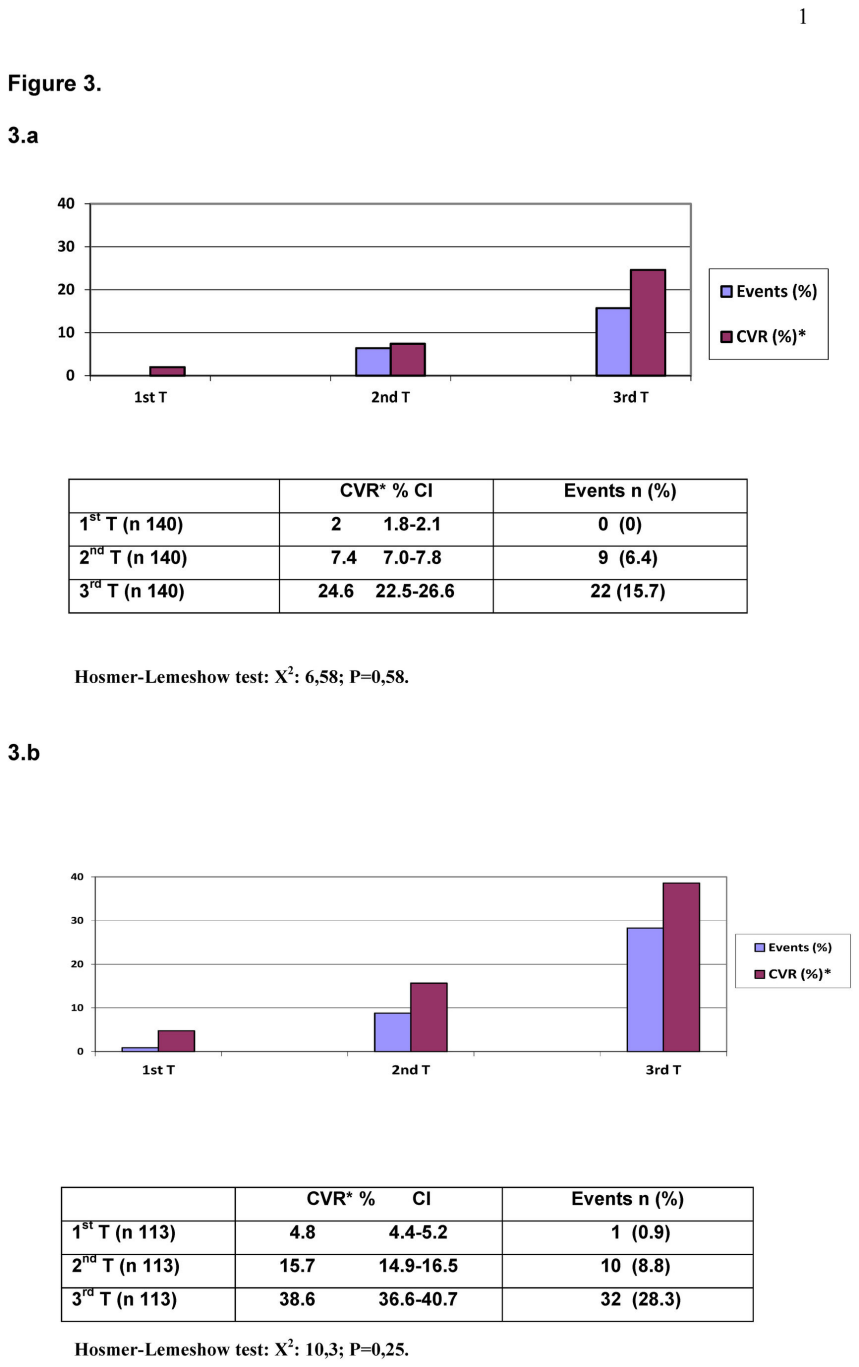
Calibration data in risk tertiles. Mean predicted CVR (%) with the D’Agostino function versus actual events over 10.6 years of follow-up in risk tertiles (3.a women and 3.b men).

**Figure 4:**
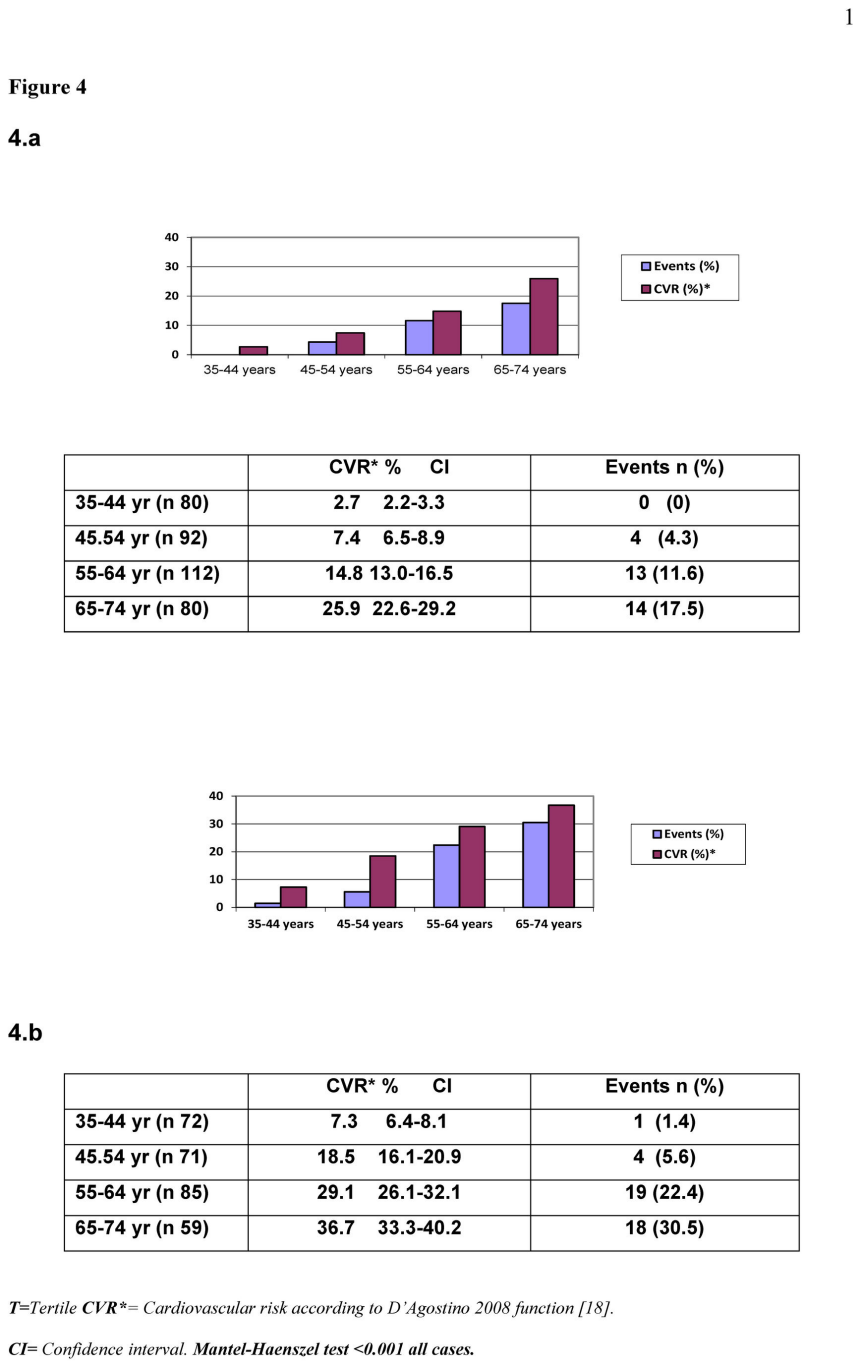
Calibration data by age-sex groups. Mean predicted CVR (%) with the D’Agostino function versus actual events over 10.6 years of follow-up by age-sex groups (4.a women and 4.b men).

Figure 3 presents the CVR in percentage and the new events in each CVR tertile and Figure 4 shows the calculated CVR versus actual events grouped according to age and sex.

There were more events the higher the risk tertile, with a clear linear trend (Mantel-Haenszel test < 0.001), such that 71.0% of the events in women and 74.4% in men occurred in the highest risk tertile (Figure 3). The calibration between the two measurements, calculated with the Hosmer–Lemeshow test was 10.3 (P=0,25) in men and 6.58 (P=0,58) in women, indicating a significant relation.

According to age and sex, Figure 4 shows that the percentage of events was close to the lowest confidence intervals of the calculated CVR, with two exceptions: older women (65-74years) and middle-aged men (45-54years).

## Discussion

Initial evaluation of the overall results suggests that applying the new “reclassification-function” cannot be recommended in our study population. The overall CVR was significantly greater in men than in women, P<0.001, whereas the events during follow-up, shown in Table 1, were 12.7% and 7.4%, respectively. In their model, D’Agostino found 18.1% and 10.1% incident events in men and women, respectively [18]. Three explanations may account for this and reduce the differences found: first, the mean follow-up periods were different, 12 years for the proposed reclassification and 10.6 years in our study, and the SD were very high in the Albacete cohort due to its small size (approximately 10% of the original-model); second, this study did not include cases of no-fatal HF as events (though they were included in the Framingham-model), which could explain an important part of the overall overestimation of the method, as there has been an increase in the incidence of HF in our area [25], and third, the levels of HDL-cholesterol were higher in the Framingham-cohort, which was not expected, though only 25% (28% in women and 22% in men–the original cohort-) of the measurements were done by precipitation, whereas 100% of our measurements were done by this method (the method available in the 1990s), and which underestimates its concentration; indeed, comparison between the old and current methods shows that the results with the new method are 10% higher, particularly when the HDL-cholesterol concentrations are low (<40 mg/dL) [26]. The CVR in our sample therefore tended to be higher.

### Discrimination of the “reclassification-model” in the sample population

Despite the above, when we wish to determine the validity [27] of the risk-function and examine its discrimination we can see that the areas under the ROC curves (Figure 2) are acceptable since the area is between 0.75 and 0.90, for both men (78.0%) and women (78.9%). They are similar to those reported by Collins and Altman for an open cohort of over a million persons, with the same function (75.2% for men and 77.0% for women, and which are comparable to those of the classic-Framingham and QRISK) [28]. Equally acceptable are the cut points for taking decisions. For example, for 75% specificity, the cut point for overall CVR is 26.6% for men (74% sensitivity) and 14.2% for women, though here with a lower sensitivity (61.3%). We should recall that these apparently high figures for CVR relate to overall CVR (prediction of any CVD, including HF).

Of particular note were the negative predictive values (Table 2) which, for both sexes, were above 80% (in low risk) and 95% (in high risk). The reading of this is that those persons in our study who were “reclassified” as having a low probability of suffering an event really experienced few events, which is an added guarantee for the physicians entrusted with their care. Examination of this same table, however, does not permit us to conclude the same for the positive predictive values, which may be of concern to health care managers, as discussed below.

### Calibration between predicted risk for CVD and new events

Examination of consistency is as important as that of discrimination when applying models derived from different populations to the study population [28]. The usual suggestion is to divide the study population into risk deciles, comparing predicted events with observed in each decile, or at least in each tertile. In our case we chose tertiles because of the sample size limitations. As expected, the events were found in the high-risk tertiles, in both men (32 of 43 events in the highest tertile, with 10 of the remaining 11 in the intermediate-risk tertile), and in women (22 of 31 events in the highest-risk tertile and the remaining 9 in the intermediate tertile).

In Figure 4, (4.a) highlights a relevant aspect when using any function to calculate CVR, i.e., the extraordinary weight of age. In our case, the theoretical population-based previsions (mean overall CVR calculated with the D’Agostino-function for each decade) slightly overestimated the risk, as occurred in the risk tertiles, but the percentage of actual events was still within the confidence intervals of the calculated CVR, assuming that the incidence of HF is similar in Albacete to the rest of Spain [25] (HF was not included at the second data collection but it was in the original model). This overestimate, therefore, is only relevant in older women (65-74 years) and middle-aged men (45-54 years), and may be partly explained by the greater documented intervention in the group of older women [19], and losses at the first study (significantly higher in active men, as usually occurs in cross-sectional studies). Nevertheless, Hosmer-Lemeshow statistic has shown a good calibration of Framingham function.

### Limitations of the study

Our sample size does not permit us to determine whether the specific calibration factors proposed in the new model for CHD, CVE and PAD (and for HF, data for which are not available) are met in our population. Nor did we use data concerning vascular age, as it is designed for individual use.

The internal validity was hardly affected by the losses (12.9%). The external validity, on the other hand, did seem to be affected by the possible changes in the original sample from the 1990s and the losses from the first cut point [21,22].

The usual biases of historical cohorts also have to be considered, particularly those relating to determining the weight of the interventions, which can only be done in part, evaluating treatment and changes in risk level. It is reasonable to suppose that this may well have been relevant.

### Future studies

Re-evaluation of this “reclassification” is needed, but replacing the analytical parameters of total cholesterol and HDL-cholesterol by the body mass index, as proposed and done by the Framingham-study researchers themselves [18]. Also necessary is the repetition of this study including HF in future cut points of the sample population. It would also be interesting to determine whether other models for evaluating CVR, particularly SCORE [9], REGICOR [11,29] and PROCAM [10], or the British models QRISK [30] and ASSIGN [31], improve the model evaluated herein. This can easily be attempted with PROCAM, which includes triglycerides and a family history of early CVD (with great weight on CVR) and which are all available for the sample. It is, however, less feasible with the other models mentioned, as the sample size in the 40-65 year age range and the fatal events are not sufficient in the case of SCORE, though it could be attempted with the British models, providing results for discussion about the controversy of whether, for low social and economic levels, the CVR is underestimated or overestimated, and vice versa in the more favored models (Matthew-effect) [32].

Finally, further information concerning the interventions and their results could be obtained by evaluating the changes in the CVR, in both older persons (with a high risk and different levels of RF) as well as in younger persons (with a low risk but certain levels of ‘treatable’ RF) [33–35].

Thus, we consider the new Framingham-proposal can be applied in our population since, in fact, the results exist despite the fact that the baseline statistics of the study cohort are completely different from those of the Framingham-cohort (Table 1). Pointing out these differences adds further value to the results that are acceptable (though not perfect), and in a practical way: cholesterol, BP, diabetes mellitus and smoking are the four most relevant RF and are sufficient for the classification of the CVR. Indeed, we believe this to be the case in populations and individuals of any origin.

In summary, although in general terms overestimation of the CVR seems to follow the usual norm for studies of populations in our area and may require calibration, as in other cases [29,30], particularly with the classic functions of Anderson (1991) [13] and Wilson (1998) [14], the statistics evaluating discrimination and consistency give more than acceptable results for the new model. Of particular note is the fact that the data for the persons reclassified as being of high risk indicate its applicability, as is the great ability of the model to detect those who will not suffer an event at all risk levels (see NPV in Table 2).

## Conclusion

This study supports the idea of using the Framingham-function recommended for primary care [18]. Despite its imperfections, it surpassed the tests of discrimination and consistency in this sample from the general population of southern-Europe (southeastern-Spain).
